# Investigating the Ordering Structure of Clustered Items Using Nonparametric Item Response Theory

**DOI:** 10.1177/00131644241274122

**Published:** 2024-09-06

**Authors:** Letty Koopman, Johan Braeken

**Affiliations:** 1 University of Groningen, The Netherlands; 2 University of Oslo, Norway

**Keywords:** coefficient *H*^*T*^, invariant cluster ordering, invariant item ordering, nonparametric item response theory, ordering structure

## Abstract

Educational and psychological tests with an ordered item structure enable efficient test administration procedures and allow for intuitive score interpretation and monitoring. The effectiveness of the measurement instrument relies to a large extent on the validated strength of its ordering structure. We define three increasingly strict types of ordering for the ordering structure of a measurement instrument with clustered items: a weak and a strong invariant cluster ordering and a clustered invariant item ordering. Following a nonparametric item response theory (IRT) approach, we proposed a procedure to evaluate the ordering structure of a clustered item set along this three-fold continuum of order invariance. The basis of the procedure is (a) the local assessment of pairwise conditional expectations at both cluster and item level and (b) the global assessment of the number of Guttman errors through new generalizations of the *H*-coefficient for this item-cluster context. The procedure, readily implemented in R, is illustrated and applied to an empirical example. Suggestions for test practice, further methodological developments, and future research are discussed.

A Guttman scale (Guttman, 1950) is an ordered set of items where a positive (e.g., correct or affirmative) response by a person to any item in the set implies that this person provided positive responses to all preceding items in the ordered set. This type of scale has attractive features in terms of interpretation (e.g., Tucker, 1953) as the meaning of a scale sum score unambiguously defines your location on the scale continuum (i.e., the last item correct in the ordered set) and determines what you can (i.e., items at earlier positions in the scale) and cannot do (i.e., items at later positions in the scale). However, a Guttman scale leaves no room for measurement error (i.e., mistakes or inconsistencies in responses), and hence, this deterministic feature makes it impossible in practice to create a working Guttman scale, except for the most trivial number of items within a narrow domain (Kofsky, 1966).

However, with minor modifications to allow for measurement error, the core principles underlying a Guttman scale have been readily adopted for test design in educational and psychological measurement: Instead of an ordered set of single items, an ordered set of item clusters may be designed; items within a cluster are considered approximately equivalent and items between clusters are considered ordered. Having clusters of items allows us to use more reliable cluster scores instead of being forced to rely on error-prone responses to a single item. Tests with such an ordered cluster structure are omnipresent in the behavioral sciences. For measuring intelligence and other abilities, such a test represents a progressive continuum that enables practitioners to track atypical development and test efficiently by for instance starting at an examinee’s age-appropriate item cluster or by stopping the test once the child is making a lot of mistakes, to not needlessly frustrate the child any further (e.g., Bishop, 1979; Dunn et al., 1997; Wechsler, 1999). Similar test formats are used for patient-reported outcomes where the item-cluster order allows for tracking the accumulation of symptoms in a severity ordering and aids in determining relevant treatment options (e.g., Dreyße et al., 2020; Roorda et al., 2005; Watson et al., 2014).

The proper functioning of these tests relies heavily on the strength of its *ordering structure*. An ordering structure means that there is in general a meaningful ordering at the cluster or item level, and that this ordering is the same for everyone (i.e., invariant). For example, if the clusters are increasing in difficulty, the first cluster should be the easiest cluster for all respondents, regardless of their individual skill levels. If, however, this is not true, then the ordering structure does not hold, which is problematic for the construct validity of the test. Hence, evaluating the ordering structure should be an essential part of investigating the construct validity of a test that is designed to measure a (theoretically) ordered construct, such as tests that reflect learning progressions or skill development (e.g., Wilson, 2009). However, most of the time there is only a tentative theoretical foundation and researchers lack the knowledge and means to validate the ordered item-cluster structure and the corresponding test administration practices and inferences that are built on that item order (Arnesen et al., 2019; Haaf et al., 2020; Huang et al., 2015; Meijer, 2010).

At the core of the validation challenge is the question whether an item (cluster) is indeed more difficult for all respondents than the preceding item (cluster) in the test. More formally, a pair of items i and j is invariantly ordered— i≼j —if the expected score on item i is lower than or equal to the expected score on item j for all values of the latent variable θ underlying the test (Sijtsma & Junker, 1996):

(1) $$i≼j:=E(X_{i}|\theta )\leq E(X_{j}|\theta ),\forall \theta .$$

The conditional expectation $E(X_{i}|\theta )$ as a function of θ is referred to as the *item response function* of item i . The “invariant” thus refers to the requirement that the order similarly applies to all individuals (regardless of their latent variable value) and hence implies that the item response functions do not intersect. An intersection would cause a reversal of item order along the latent dimension. The invariant ordering property is not deterministic as with a Guttman scale, but stochastic as implied by the expected values.

If pairwise invariant ordering (Equation 1) holds for all items in an item set, the ordering structure of the item set is an *invariant item order* (IIO). Yet, an IIO is an unrealistic requirement for various scales (Meijer & Egberink, 2012; Sijtsma & Van der Ark, 2022), and not equipped to handle scales with clustered items. Hence, there is a practical need for ordering properties that are less restrictive compared to IIO, but which nevertheless provide information on the ordering structure of a scale. One promising way to achieve this may be by focusing on the ordering of clusters of items rather than the ordering of individual items (Ligtvoet et al., 2011; Van der Ark & Van Diem, 2003). However, incorporating the cluster level is challenging for three reasons: First, it is not clear whether a definition of an *invariant cluster ordering* (ICO) should relate to the expectation of the cluster scores or to the expectation of item scores across clusters; second, a structured approach is lacking to distinguish violations at the item level within and between clusters, but also violations at the cluster level; and third, no overall coefficients exist to provide information on whether an ordering is better represented at the item level or at the cluster level.

In this article, we develop a procedure to evaluate the ordering structure of scale with fixed item clusters, based upon initial work in nonparametric item response theory (IRT; Ligtvoet et al., 2010; Sijtsma & Junker, 1996). In the process, we conceptualize different levels of invariant item and cluster orderings, suggest local evaluation methods of discrepancies among empirical response functions, and propose a multilevel extension of the $H^{T}$ coefficient for global evaluating of the ordering consistency (Sijtsma & Meijer, 1992). The procedure is applied to test data from the Norwegian version of the Test for Reception of Grammar (TROG; Bishop, 1979). Suggestions for practice, further methodological developments, and future research are discussed.

## Ordering Structures of Clustered Items

Let an item set consist of J items, indexed i or j (i,j=1,2,…,J;i≠j) . Let $X_{i}=x$ denote the response on item i which can take one of the m+1 -ordered categories (x=0,…,m) . As mentioned, the item response function is the expected value for the item score as a function of the latent variable θ :

(2) $$E(X_{i}|\theta )=\sum_{x=1}^{m}\mathrm{Pr}(X_{i}\geq x|\theta ).$$

Let the items be divided into C mutually exclusive clusters, indexed c or d (c,d=1,2,…,C;c≠d) . Each cluster c consists of $J_{c}$ items, and $\sum_{c=1}^{C}J_{c}=J$ . The set $\Omega_{c}$ represents all items part of cluster c , and we use $X_{c(i)}$ to denote the response on item i part of cluster c . This extra structure on the items implies that, beyond score variation at the item level, we can now also further distinguish between score variation within an item cluster and between-item clusters.

Let the *cluster response function* be the expected value of the average within-cluster item score:

(3) $$E(X_{c(\cdot )}|\theta )=J_{c}^{-1}\sum_{i\in \Omega_{c}}E(X_{c(i)}|\theta ).$$

Let a clustered item set be ordered and numbered according to increasing $E(X_{c(\cdot )})$ . A pair of clusters is invariantly ordered— c≼d —*at cluster level* if the expected score on cluster c is lower than or equal to the expected score on cluster d for all values of the latent variable θ :

(4) $$c≼d:=E(X_{c(\cdot )}|\theta )\leq E(X_{d(\cdot )}|\theta ),\forall \theta .$$

This pairwise ICO at the cluster level has only weak implications, as it does not exclude violations of item ordering at the item level. If, for a clustered item set, Equation 4 holds for all cluster pairs for which c<d , the ordering structure of the item set is a *weak ICO*.

A pair of clusters is invariantly ordered *at item level* if the expected score on each item i in cluster c is lower than or equal to the expected item score on each item j in cluster d for all values of the latent variable θ :

(5) $$c≼d:=E(X_{c(i)}|\theta )\leq E(X_{d(j)}|\theta ),\forall i\in \Omega_{c},\forall j\in \Omega_{d},\forall \theta .$$

This pairwise invariant ordering at item level has strong implications, as it requires ordering at the cluster level and ordering at the item level for items across different clusters. If, for a clustered item set, Equation 5 holds for all item pairs with c<d , the ordering structure of the item set is a *strong ICO*.

If, for a clustered item set, pairwise IIO (Equation 1) holds for all items in an item set, the *ordering structure* of the item set is an IIO. An IIO has strong implications, as it requires ordering at the cluster level, ordering of the items across different clusters, and ordering of the items within clusters.

**Figure 1:**
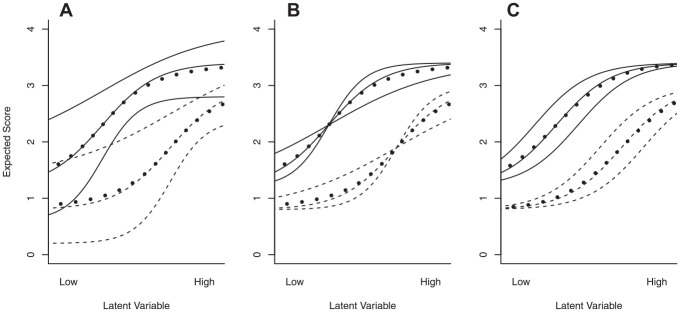
Three Examples of Ordering Structures for Two Clusters of Three Items. Based on the Intersection of the Item Response Functions (Solid for Items in the First Cluster and Dashed for Items in the Second Cluster) and Cluster Response Functions (Dotted), the Item Set Has a (a) Weak ICO, (b) Strong ICO, or an (c) IIO

The difference between the weak ICO, strong ICO, and IIO is illustrated in Figure 1. Weak ICO (panel (a)) only requires cluster response functions to not intersect and does not impose any restrictions at the item level, whereas strong ICO (panel (b)) requires all item response functions across different clusters not to intersect, but has no such restrictions for item response functions within the same cluster. IIO (panel (c)) requires all item response functions within and between clusters not to intersect. IIO implies strong ICO, which implies weak ICO, but not vice versa; except for the trivial case where C=J and all clusters consist of a single item, in which case we would simply speak of an IIO.

### Quantifying the Ordering Structure

With its focus on ordering of persons and ordering of items, nonparametric IRT has been shown to provide a useful framework for investigating whether a test (with nonclustered items) has an IIO (Sijtsma & Hemker, 1998; Sijtsma & Junker, 1996). Within this framework, the most general approach to study IIO, applicable to both dichotomous and polytomous items, was proposed by Ligtvoet et al. (2010), based upon pairwise evaluation of item response functions. Given that an IIO has been established, the overall consistency of the item ordering by respondents can be evaluated with coefficient $H^{T}$ (Sijtsma & Meijer, 1992). The existing methods are not suitable for clustered item, because they do not distinguish violations at the item level within and between clusters, nor do they recognize violations at the cluster level. In addition, no overall coefficients exist to provide information on whether an ordering structure exist at the cluster level nor whether the ordering is better represented at the item level or at the cluster level. After briefly describing the exploratory procedure for investigating an IIO in the case of nonclustered items, we describe how the same core principles can be adapted to enable an investigation of the ordering structure of clustered items. Throughout, we will refer to the pairwise evaluation as *local fit* and to overall consistency as *global fit*.

### Methods for Nonclustered Items

#### Local Fit

The procedure by Ligtvoet et al. (2010) is exploratory and involves an iterative heuristic search for an item set that has an IIO. First, items are ordered and numbered according to their manifest marginal item means ${\bar{X}}_{i}$ . Second, the (latent) IIO property of Equation 1 is translated to a manifest IIO property, by replacing latent variable θ by manifest proxy variable R , which is independent of the two items of focus i and j ; a natural choice is the rest score $R_{\left(i,j\right)}=X_{+}-X_{i}-X_{j}$ (i.e., the sum score across all items excluding the item pair). This results in the property of manifest IIO:

(6) $$i≼j:=E(X_{i}|R_{\left(i,j\right)}=r)\leq E(X_{j}|R_{\left(i,j\right)}=r),\forall i<j,\forall r.$$

For a locally independent item set, IIO implies manifest IIO (Ligtvoet et al., 2011, Corollary). The expected item scores in Equation 6 are then estimated by their sample equivalents ${\bar{X}}_{i|r}$ (i.e., the mean score of item i in rest-score group r ). This formulation opens up for a series of one-sided significance tests of the directional null hypothesis along the rest-score values r : $H_{0}:{\bar{X}}_{i|r}-{\bar{X}}_{j|r}\leq 0$ (a hypergeometric test for dichotomous items or a one-sided dependent t -test for polytomous items; see Molenaar & Sijtsma, 2000, pp. 71–72, and Ligtvoet et al., 2010, respectively). A significant test outcome for at least one of the rest-score values is evidence supporting a violation of the IIO for that item pair. The most heavily affected item (i.e., the item that is involved in the most violations of pairwise IIO) is eliminated from the item set, and everything is reiterated until an item set remains for which an IIO holds.

#### Global Fit

For an item set that has an IIO, coefficient $H^{T}$ is estimated as a global index for how consistent the items are ordered by respondents (Ligtvoet et al., 2010; Sijtsma & Meijer, 1992). This measure is the Mokken scalability coefficient H (Loevinger, 1948; Mokken, 1971, p. 174) applied to the transposed persons by items test data matrix. Let N respondents be indexed n or *o* (n,o=1,2,…N;n≠o) . Let ${\mathrm{Cov}}_{\mathrm{no}}^{W}$ denote the covariance of the scores of respondents n and o within items. In addition, let ${\mathrm{Cov}}_{\mathrm{no}}^{W}(\max)$ denote the maximum covariance between the scores within items, given the marginal distributions of respondents n and o . Coefficient $H^{T}$ is defined as:

(7) $$H^{T}=\frac{\sum \sum_{n<o}^{N}{\mathrm{Cov}}_{\mathrm{no}}^{W}}{\sum \sum_{n<o}^{N}{\mathrm{Cov}}_{\mathrm{no}}^{W}(\max)}.$$

$H^{T}$ can also be written in terms of errors patterns according to a Guttman scale (Sijtsma & Meijer, 1992). Such an error occurs if a more able person n fails an item, whereas a less able person o passes the same item. For a perfect Guttman scale, no such errors occur, resulting in $H^{T}=1$ .

For a locally independent item set that satisfies an IIO, $0\leq H^{T}\leq 1$ , with higher values indicating a more consistent ordering of the items by the respondents, that is, a stronger agreement of respondents’ item-score patterns with the item ordering in the total sample and a stronger resemblance to a Guttman scale. All other things being equal, the magnitude of $H^{T}$ is higher for item sets in which the item response functions are further apart or steeper. Note that $H^{T}$ may take on positive values for sets with intersecting item response functions. Hence, $H^{T}$ should not be used to determine whether an item set satisfies an IIO. Rather, given that an IIO has been established, coefficient $H^{T}$ gives an indication of how strong this ordering is.

### Generalizations for Clustered Items

Rather than an exploratory approach that uses the empirical item means to order and number the items, a more confirmatory approach may consider an a priori defined, theoretical ordering of clustered items. If no a priori ordering is defined within clusters, we suggest ordering and numbering items within each cluster based on their mean scores. The goal is to assess the structure (local fit) and strength (global fit) of the assumed ordering. In increasing order of strictness, the ordering structures to be assessed for clustered items are weak ICO, strong ICO, and IIO.

#### Local Fit

The basis for investigating weak and strong ICO will be corresponding adaptations to the comparison of item response functions as given in Equation 6. To enable between-cluster investigation, we propose using a cluster rest score $R_{\left(c(\cdot ),d(\cdot )\right)}$ , which is the sum score of all cluster scores excluding cluster c and d . To enhance comparability of estimated item and cluster response functions, we suggest using rest score $R_{\left(c(\cdot ),d(\cdot )\right)}$ at both the cluster and item levels for evaluating the ordering structure. Weak ICO implies an investigation at cluster level and will be accommodated by investigating the manifest versions of the corresponding cluster response functions. This results in manifest weak ICO:

(8) $$c≼d:=E(X_{c(\cdot )}|R_{\left(c(\cdot ),d(\cdot )\right)}=r)\leq E(X_{d(\cdot )}|R_{\left(c(\cdot ),d(\cdot )\right)}=r),\forall c<d,\forall r.$$

In contrast, strong ICO requires an investigation at item level and will be accommodated by investigating the manifest order property as in Equation 6, with the modification that an order violation can only occur for items pertaining to a different item cluster. This results in manifest strong ICO:

(9) $$c≼d:=E(X_{c(i)}|R_{\left(c(\cdot ),d(\cdot )\right)}=r)\leq E(X_{d(j)}|R_{\left(c(\cdot ),d(\cdot )\right)}=r),\forall c<d,\forall r.$$

To evaluate IIO for clustered items, we suggest replacing rest score $R_{\left(i,j\right)}$ with $R_{\left(c(\cdot ),d(\cdot )\right)}$ in Equation 6, with c and d being the clusters to which items i and j belong, respectively (if c=d , $R_{\left(c(\cdot )\right)}$ is used; i.e., the sum score on all cluster scores, excluding cluster c ). Hence, evaluating manifest IIO in the presence of item clusters entails evaluating manifest strong ICO (which equals manifest IIO of item pairs across clusters) plus evaluating manifest IIO (which additionally requires manifest IIO of item pairs within the same cluster).

#### Global Fit

**Figure 2:**
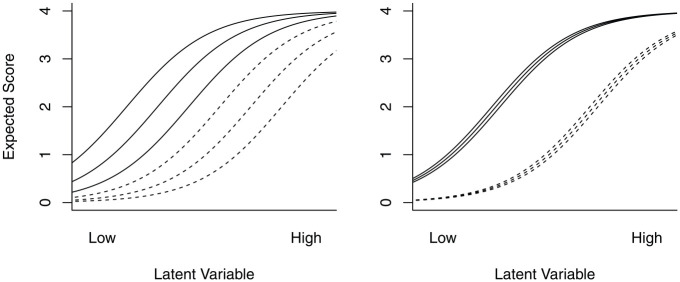
Left Panel: Two Clusters of Three Item Response Functions With Equidistant Difficulty. Right Panel: Two Clusters of Three Item Response Functions With Similar Difficulty. In Both Cases, Coefficient $H^{T}=.47$

The practical use of coefficient $H^{T}$ is limited in the case of clusters of items, even if IIO holds. For example, $H^{T}$ does not distinguish between, say, six items that are equidistant in difficulty (i.e., clearly ordered, see Figure 2, left panel) compared to two clusters of three items each, for which the clusters are strongly ordered, but where the item response functions within clusters are very similar (see Figure 2, right panel). For both situations in Figure 2, $H^{T}=.47$ and visualization is the only way of distinguishing between the two situations (Meijer & Egberink, 2012).

We aim at distinguishing between the ordering consistency of items and the ordering consistency of clusters. This can be achieved by evaluating not only the consistency of respondent scores *within items* but also the consistency of respondent scores *between items* within the same cluster. We approach this distinction using a similar strategy as Snijders (2001), who generalized H to clusters of respondents (see also Crişan et al., 2016; Koopman et al., 2020, 2022). Let ${\mathrm{Cov}}_{\mathrm{no}}^{B}$ denote the covariance of the scores of respondents n and o between different items within the same cluster. In addition, let ${\mathrm{Cov}}_{\mathrm{no}}^{B}(\max)$ denote the maximum covariance of the between-item scores, given the marginal distributions of respondents n and o . Then, coefficient $H^{\mathrm{TB}}$ is defined as:

(10) $$H^{\mathrm{TB}}=\frac{\sum \sum_{n<o}^{N}{\mathrm{Cov}}_{\mathrm{no}}^{B}}{\sum \sum_{n<o}^{N}{\mathrm{Cov}}_{\mathrm{no}}^{B}(\max)}.$$

$H^{\mathrm{TB}}$ can also be written in terms of errors patterns according to a Guttman scale. Such an error occurs if a more able person n fails one item in a cluster, whereas a less able person o passes a different item in that cluster. For a perfect cluster-level Guttman scale, no such errors occur, resulting in $H^{\mathrm{TB}}=1$ . Note that $H^{\mathrm{TB}}$ can only be computed on clusters that contain at least two items. Hence, if a cluster has only one item, it will be ignored in the computations. Koopman et al. (2020) showed that point estimates of these coefficients for clusters of respondents (rather than clusters of items) were unbiased for a wide range of conditions, including number of clusters and cluster size. It is expected that results are similar for clusters of items.

$H^{\mathrm{TB}}$ is a measure for the consistency of how the respondents order the clusters. For a locally independent clustered item set satisfying a (weak or strong) ICO or IIO, $H^{\mathrm{BT}}\geq 0$ . Because cluster response functions are flatter than the item response functions, and because the range of the difficulty for the clusters is more limited than for the items, $H^{\mathrm{TB}}\leq H^{T}$ (see the Appendix of Snijders, 2001). Hence, coefficient $H^{\mathrm{TB}}$ is an attractive choice for distinguishing between item- and cluster ordering, because it is directly comparable to $H^{T}$ . The ratio $H^{\mathrm{TB}}/H^{T}$ may be used as indication whether the ordering in the test is at the item level or at the cluster level. If $H^{\mathrm{TB}}=H^{T}$ , $H^{\mathrm{TB}}/H^{T}=1$ , meaning that the ordering structure in the item set is fully accounted for by the cluster ordering. If $H^{\mathrm{TB}}=0$ , $H^{\mathrm{TB}}/H^{T}=0$ , and there is no meaningful ordering present at the cluster level. For Figure 2, $H^{T}=.47$ for both panels. However, for the left panel $H^{\mathrm{TB}}=.33$ and $H^{\mathrm{TB}}/H^{T}=.69$ , whereas for the right panel, $H^{\mathrm{TB}}=.47$ and $H^{\mathrm{TB}}/H^{T}=.99$ . For both situations, there is a substantial cluster ordering. However, in the left panel, there is also unique item ordering within the clusters, whereas in the right panel, the cluster ordering explains virtually all the ordering in the item set.

As tentative guidelines, we propose the following. First, we suggest, based on Ligtvoet et al. (2010), to interpret $H^{T}$ as a general measure of the consistency of the ordering structure, using the guidelines in Table 1. Note that both types of ICO allow for specific types of IIO violations that can temper the magnitude of $H^{T}$ . Second, we suggest, based on Snijders (2001), to evaluate ratio $H^{\mathrm{TB}}/H^{T}$ as the consistency of the ordering structure at the cluster level, using the guidelines in Table 2. We avoid only interpreting the ratio, because the ratio can be high even when $H^{T}$ is close to zero, in which case there is still no meaningful ordering in the ordering structure (for similar baseline concerns with ratio indices, see, e.g., Van Laar & Braeken, 2022). Note that both $H^{T}$ and $H^{\mathrm{TB}}$ can be positive in the absence of an ordering structure. Hence, although for a meaningful ordering structure these coefficients should be sufficiently high; they are not sufficient to conclude an ordering structure in itself and thus should only be interpreted after establishing the ordering structure through local fit evaluation.

**Table 1:** Guidelines for Interpreting $H^{T}$ as the Consistency of the Ordering Structure

$H^{T}<.30$ : too inconsistent to be useful
$.30\leq H^{T}<.40$ : low consistency
$.40\leq H^{T}<.50$ : medium consistency
$H^{T}\geq .50$ : high consistency

*Note*. Ordering interpretation is only valid for an established ordering structure.

**Table 2:** Tentative Guidelines for Interpreting Ratio $H^{\mathrm{TB}}/H^{T}$ as Consistency of Cluster Ordering

$H^{\mathrm{TB}}/H^{T}<.3$ : too inconsistent to be useful
$.3\leq H^{\mathrm{TB}}/H^{T}<.6$ : reasonable consistency
$H^{\mathrm{TB}}/H^{T}\geq .6$ : high consistency

*Note*. Ordering interpretation is only valid for an established ordering structure.

## Investigating the Ordering Structure of a Clustered Item Set

**Figure 3:**
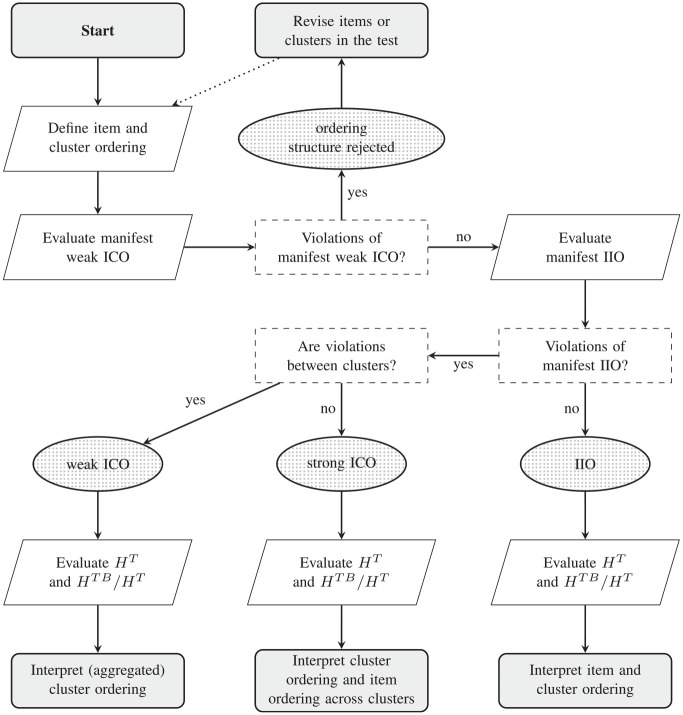
Flowchart of the Procedure for Investigating the Ordering Structure for a Given Set of Clustered Items *Note*. IIO = Invariant Item Ordering, ICO = Invariant Cluster Ordering.

Figure 3 shows a flow chart of a structural procedure for investigating the ordering structure of a test with a given clustering of the items.

The procedure commences with defining the cluster ordering for the population. This ordering may be based on theory or estimated from the sample data (e.g., by ordering the clusters from easy to difficult). If desired, an item ordering (within clusters) may also be defined. If only a cluster ordering is defined, items within clusters are assumed unordered. This means that these items may be equally difficult or violating IIO. Manifest weak ICO is evaluated for all cluster pairs. If significant violations of manifest weak ICO are present, any ordering structure is rejected, because violation of weak ICO implies violation of strong ICO and of IIO for item clusters. Note that this does not mean there exists no ordering structure in the test, just that the investigated structure does not hold for the a priori defined item and cluster order.

If no violations exist, manifest IIO for item clusters is investigated, as this entails both manifest strong ICO and manifest IIO of item pairs within clusters. If significant violations of manifest IIO are revealed between clusters, it is concluded that only weak (and not strong) ICO holds for the test. Coefficient $H^{T}$ is used to evaluate whether there is any meaningful ordering structure in the total item set using the guidelines in Table 1. If yes, ratio $H^{\mathrm{TB}}/H^{T}$ is used to evaluate the consistency of the cluster ordering, using the guidelines in Table 2. If $H^{\mathrm{TB}}/H^{T}$ is sufficiently large, the ordering structure of the test may be interpreted at the cluster level. Item-level interpretations of the clustering should be done with care, as the ICO only holds for the aggregated cluster level, but not for (all) individual items.

If significant violations are only present within clusters, it is concluded that a strong ICO holds for the test. Coefficient $H^{T}$ is used to evaluate whether there is any meaningful ordering structure in the total item set using the guidelines in Table 1. If yes, ratio $H^{\mathrm{TB}}/H^{T}$ is used to evaluate the consistency of the cluster ordering, using the guidelines in Table 2. If $H^{\mathrm{TB}}/H^{T}$ is sufficiently large, the ordering structure of the test may be best interpreted at the cluster level. Otherwise, the ordering structure is best interpreted at the item level, but only across clusters.

If no significant violations of manifest IIO are revealed, it is concluded that an IIO holds for the test. Coefficient $H^{T}$ is used to evaluate the consistency of the item ordering, using the guidelines in Table 1. Ratio $H^{\mathrm{TB}}/H^{T}$ is used to evaluate the consistency of the cluster ordering, using the guidelines in Table 2. If $H^{\mathrm{TB}}/H^{T}$ is large, the ordering structure of the test is best interpreted at the cluster level. Otherwise, the ordering structure is best interpreted at the item level.

Revising the items, the clustering, their defined order, or removal of items or clusters may result in a (stronger) ordering structure. The output from the analysis indicated which items or clusters are problematic. For example, clusters or items from clusters may be subsequently removed, until a test satisfies a desired ordering structure. However, revisions and removing items may substantially affect the empirical cluster ordering and may invalidate previously drawn conclusions pertaining the weak ICO structure. Be aware that such an iterative procedure comes with all the risks and no guarantees similar to what applies to (mis)specification searches in other psychometric contexts (see, e.g., MacCallum, 1986).

## Real-Data Example

As an illustration, we will look at item response data of N=210 children (105 girls and 105 boys; measurement taken at a first time point of a larger developmental study; see Brinchmann et al., 2019) that took the Norwegian adaptation of the TROG (Bishop, 1979). The TROG consists of C=20 clusters of $J_{c}=4$ dichotomously scored items, and all children were administered each of the J=80 items. The items in each subsequent cluster are intended to reflect an increasingly difficult grammatical structure, but items within a cluster are intended to have similar complexity. Thus, theoretically, this aligns with the strong ICO assumption. To our knowledge, no formal invariant ordering structure analysis has been performed on the TROG nor on the Norwegian language adaptation that we will study.

We investigated the ordering structure of the TROG using the procedure displayed in Figure 3. We implemented the proposed methodology in the check.iio() function from the R-package mokken (Van der Ark, 2007, 2012), in which the data are also included under the name trog. This function evaluates manifest weak ICO (based on Equation 4) and evaluates manifest IIO for item clusters using the cluster-level rest scores from Equation 9, rather than the item-level rest scores from Equation 6, while distinguishing between violations within and between clusters. In this function, adjacent rest-score groups are combined until a minimum size is satisfied in order to avoid instable proportion estimates (i.e., minsize argument of the function; see, e.g., Molenaar & Sijtsma, 2000, p. 67). For this data set, the minimum size for the rest-score groups defaulted to 70, which resulted in two rest-score groups in 99% of the pairwise comparisons, and 3 rest-score groups otherwise. Conditional differences between rest-score groups were flagged, in line with the current default procedure, when the difference in proportion was at least .03 (i.e., minvi argument of the function) and were evaluated at the 5% significance level (i.e., alpha argument of the function). Syntax files are available to download from the Open Science Framework via https://osf.io/xn56t.

### Descriptives

**Figure 4:**
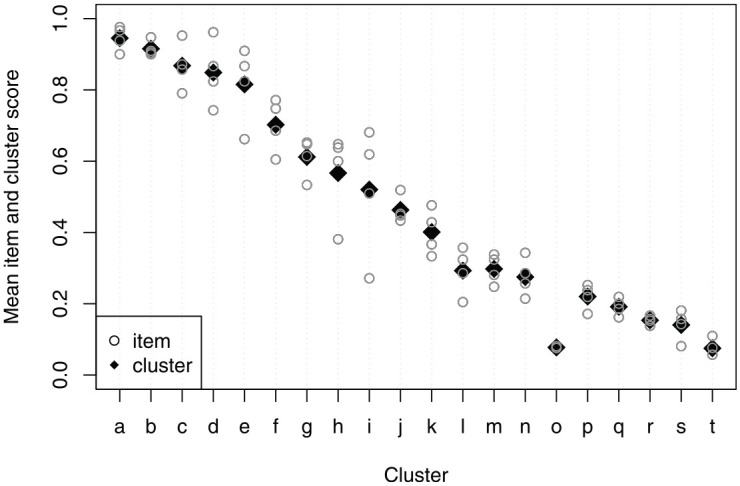
Observed Mean Item Scores (Circles) and Mean Cluster Scores (Diamonds) for the 20 Theoretically Ordered Clusters (4 Items per Cluster) of the TROG in the Total Sample

Figure 4 shows the mean item and cluster scores for the theoretically defined cluster ordering. In general, the defined cluster order coincides with the empirical cluster order (i.e., the order in mean cluster scores), but two clusters may be displaced. Cluster l has a slightly lower mean $\left({\bar{X}}_{l\cdot }=0.29\right)$ than cluster m $\left({\bar{X}}_{m\cdot }=0.30\right)$ , whereas, cluster o has a substantially lower mean $\left({\bar{X}}_{o\cdot }=0.08\right)$ than clusters p $\left({\bar{X}}_{p\cdot }=0.22\right)$ to s $\left({\bar{X}}_{s\cdot }=0.14\right)$ . This is an early indication that the defined cluster order may be inappropriate. Figure 4 also shows a substantial variation of mean item scores within some of the clusters, with the most prominent in cluster i . This is an indication that the items are not similarly difficult within the same cluster, making strong ICO unlikely. Note that Figure 4 is not informative on whether the cluster ordering is invariant, as it reflects the average ordering across all respondents.

### Investigating the Ordering Structure

#### Local Fit

Manifest weak ICO was for each cluster pairwise evaluated with the other 19 clusters. Table 3 summarizes the local fit evaluation of the test’s theoretical ordering structure and shows for each cluster for how many cluster pairs there was at least one significant violation of manifest weak ICO (Column 3), the largest violating distance (i.e., the largest absolute distance between the indices of the violating cluster pairs; Column 4), and the value of the largest violation (i.e., the maximum observed pairwise difference between conditional cluster means; Column 5). There was at least one significant violation of manifest weak ICO for cluster o with four other clusters (Table 3). In this case, it is possible to deduce that cluster o violates with cluster p,q,r, and s , as cluster o violates with four clusters, whereas the four subsequent clusters violate only with one other cluster, hence that needs to be o . Based on these results, the ordering structure of the theoretically defined cluster order is rejected.

**Table 3:** The Number of Clusters Pairs the Cluster Is Involved in Violating Manifest Weak ICO (#vipairs), the Largest Violating Distance ( $D_{c}$ ), and the Value of the Largest Violation (Maxvi) for the Theoretical Cluster Order

Index	Cluster	Weak ICO		
		#vipairs	$D_{c}$	maxvi
1	a	0	0	.00
2	b	0	0	.00
3	c	0	0	.00
4	d	0	0	.00
5	e	0	0	.00
6	f	0	0	.00
7	g	0	0	.00
8	h	0	0	.00
9	i	0	0	.00
10	j	0	0	.00
11	k	0	0	.00
12	l	0	0	.00
13	m	0	0	.00
14	n	0	0	.00
15	o	4	4	.22
16	p	1	1	.22
17	q	1	2	.18
18	r	1	3	.12
19	s	1	4	.10
20	t	0	0	.00

**Figure 5:**
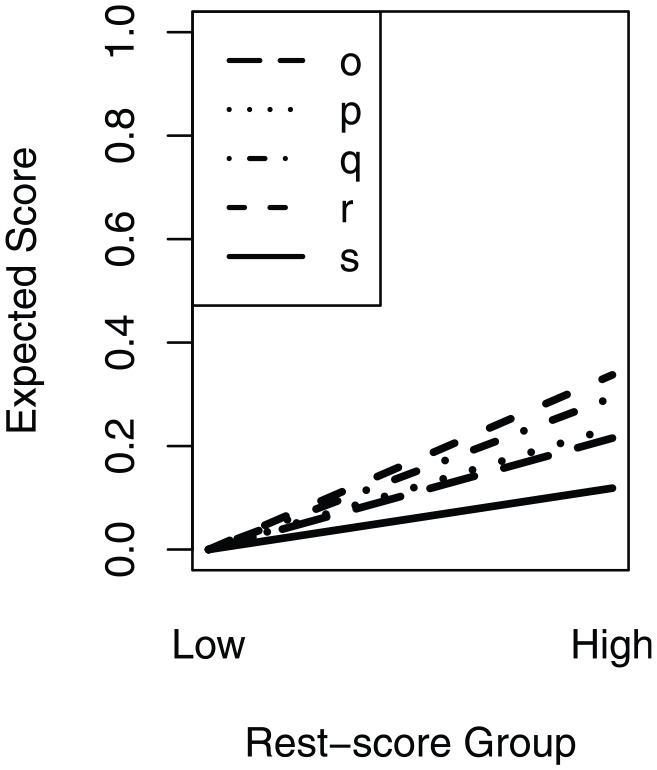
Estimated Cluster Response Functions of Clusters o,p,q,r, and s

Figure 5 shows the estimated cluster response functions for clusters o,p,q,r, and s , to allow for inspection of the violations. The results showed no difference in mean cluster score in the first rest-score group and a significant deviation from the theoretically defined order in the second rest-score group. The result that cluster o was as difficult or more difficult than clusters p , q , r , and s for all rest-score groups provides evidence that the theoretical position of cluster o is incorrect, instead of the ordering of the clusters not being invariant. Hence, we changed the index position of cluster o from 15 to 19, which also changed the index of clusters p to s (see Table 4, first two columns). Note that we maintained the theoretical order of cluster l and m , as the reversed empirical order did not significantly violate manifest weak ICO.

**Table 4:** Number of Violating Clusters (Item) Pairs the Cluster (Item) Is Involved in (#vipairs), the Largest Violating Distance $\left(D_{c}\right)$ , and the Value of the Largest Violation (Maxvi), for Manifest Weak ICO and Manifest Strong ICO, for the Redefined Cluster Order

Index	Cluster	Weak ICO	Strong ICO			
		# ${\mathrm{vipairs}}_{c}$	# ${\mathrm{vipairs}}_{i}^{B}$	# ${\mathrm{vipairs}}_{c}$	$D_{c}$	maxvi
1	a	0	3	3	3	.11
2	b	0	5	2	2	.17
3	c	0	7	2	2	.31
4	d	0	14	4	5	.31
5	e	0	8	3	4	.23
6	f	0	4	2	3	.23
7	g	0	9	2	2	.29
8	h	0	15	3	3	.44
9	i	0	30	5	5	.44
10	j	0	8	2	2	.35
11	k	0	6	2	3	.33
12	l	0	10	3	3	.22
13	m	0	7	2	4	.21
14	n	0	7	3	5	.22
15	p	0	2	2	3	.09
16	q	0	1	1	1	.07
17	r	0	1	1	1	.07
18	s	0	1	1	1	.07
19	o	0	0	0	0	.05
20	t	0	0	0	0	.05

*Note*. $D_{c}$ and maxvi have been omitted for weak ICO, as they were zero for all clusters.

Evaluating manifest weak ICO for the redefined cluster order showed no significant violations of manifest weak ICO (Table 4, Column 3). This confirms that the violations in the first manifest weak ICO analysis were explained by the cluster not being in the appropriate position, rather than the cluster ordering not being invariant.

We continued the analysis by evaluating manifest IIO for item clusters, as this includes evaluating strong ICO and IIO. Here, we merely focus on the results relevant to strong ICO; hence, manifest IIO was evaluated for each item with the 76 items that were in a different cluster (between), ignoring the three items that were in the same cluster (within). Across all J(J−1)/2=3,160 possible item pairs, only those comparisons between items belonging to different clusters are relevant to evaluate strong ICO. For a set of 20 clusters with four items each, this resulted in a total of $CJ_{c}(J-J_{c})/2=20\times 304/2=3,040$ relevant between-cluster pairwise item comparisons, and $CJ_{c}(J_{c}-1)/2=20\times 12/2=120$ ignored within-cluster pairwise item comparisons. Table 4 shows for each cluster in how many significant item-pair violations of manifest strong ICO the items in the clusters are involved (# ${\mathrm{vipairs}}_{i}^{B}$ ; i.e., violations between clusters, Column 4), distributed over how many unique clusters (# ${\mathrm{vipairs}}_{c}$ ; Column 5), the largest violating distance ( $D_{c}$ ; Column 6), and the value of the largest violation (maxvi; Column 7). There are numerous violations of manifest strong ICO, especially for cluster i . Combined, the items in cluster i are involved in 30 item pairs that significantly violate manifest strong ICO, which is about 10% of the total number of comparisons, distributed over five unique clusters. The furthest cluster that contains an item that violates with an item in cluster i is five clusters away. The largest violation between two items was .44. Note that for 15 clusters, strong ICO was only violated with items that were at most three clusters away. Because the number of violations of manifest strong ICO is so numerous and because Figure 4 shows substantial variation in mean item scores within many clusters, we believe achieving a strong ICO is infeasible without the redesign or removal of many items. Hence, we avoid further exploration of removing/reordering clusters and reject a strong ICO for the given (redefined) cluster order, which implies that an IIO is also rejected. In an Online Supplement, we provide some item-level results to illustrate how to pursue such an exploration (available from the Open Science Framework via https://osf.io/xn56t).

#### Global Fit

Based on the results, we conclude that the ordering structure of the TROG with the redefined order is a weak ICO. The general consistency of the ordering structure of the test was evaluated with $H^{T}$ and $H^{\mathrm{TB}}/H^{T}$ , using the guidelines in Table 2. First, $H^{T}=0.69$ , indicating a high consistency of ordering in the data. Second, $H^{\mathrm{TB}}/H^{T}=0.95$ , indicating that this consistency is almost entirely explained by the clustering.

A weak ICO allows for interpreting the ordering structure at the cluster level, but not at the individual-item level. Hence, even though the clusters are increasing in difficulty, individual items may be more or less difficult than expected based on their cluster. The weak ICO ordering structure supports the practice of administering the test in the theoretical cluster order, for which cluster o is moved to the penultimate position. Note that the sample size was smaller than desired for an ordering structure analysis (e.g., Straat et al., 2014; Watson et al., 2018; Wind, 2022). As a result, in general, only two rest-score groups were formed for item-pair and cluster-pair comparison. This limits the estimation of the item response function, because the item means are only estimated for two groups, possibly averaging out less obvious violations of manifest weak ICO. Hence, these results are tentative and should be interpreted with caution.

## Discussion

For clustered items, an invariant ordering can apply at various levels and in various degrees of strictness. Furthermore, a distinction needs to be made between ordering of items *within* a cluster, where no a priori expected order restrictions apply, and the ordering of items *between* clusters, where an ordering is required. Evaluating the ordering structure of a test benefits the interpretation of a test and test scores and is a requirement to allow various types of test administration, including presenting items in ascending difficulty, and using starting and stopping rules in a sequentially ordered test. In this article, we distinguished three increasingly strict types of ordering to describe the ordering structure of a measurement instrument with item clusters. Weak ICO requires an invariant ordering defined at the aggregate level of cluster scores, whereas strong ICO requires the invariant ordering to apply directly at the item-score level between all items belonging to different clusters. IIO is the strongest property, requiring all items to be invariantly ordered, between but also within clusters.

Building on nonparametric IRT, we proposed a procedure for evaluating the ordering structure of a clustered item set according to these sequentially stricter invariant ordering properties. These properties are investigated by evaluating violations of manifest weak ICO for all cluster pairs and manifest IIO for all item pairs, where the latter distinguishes between within- and between-cluster violations. To support this investigation of local ordering violations, we introduced coefficient $H^{\mathrm{TB}}$ to be directly compared to existing coefficient $H^{T}$ , as a more global effect size measure to quantify the consistency of the observed ordering structure.

Besides being intuitive and flexible, the procedure is also computational straightforward, making it an attractive tool for practitioners in the field that are concerned with the validity of the ordering structure of their measurement instrument. For even better accessibility, we implemented the methodology into function check.iio of package mokken in open statistical software environment R (R Core Team, 2023). The data are also included in this package under the name trog (e.g., syntax, see the Open Science Framework via https://osf.io/xn56t).

The suggested analysis is confirmatory in nature, useful to evaluate the ordering structure of a fixed clustering of items, after which the ordering or clustering may be manually adapted. Hence, this approach is an important part of investigating the construct validity of an instrument that in theory has an ordering structure. Future simulation studies can provide insight how the method performs in the presence of different types and severity of violations and different cluster characteristics. Furthermore, they may update the suggested guidelines for the global fit coefficients. In addition, items may not naturally be clustered, requiring a more exploratory approach. In that case, items may be ordered based on their observed item means, and method manifest IIO may then be manipulated to combine items into clusters until the desired ordering structure holds (also, see Van der Ark & Van Diem, 2003). From the latter perspective, generalizations of the approach toward a more exploratory detection of item clusters, weighting violations between items based on neighborhood proximity, could be of interest for future work. Simulation studies could contribute to evaluating the performance of different methods, how (well) clusters are constructed, and the preferred number of clusters, items, and respondents. Computationally, the proposed methods work if there are at least three clusters containing at least two items each (although the number of items may vary across clusters). Moreover, future research can investigate the relation between the ordering structure of a test and guidelines for adaptive test administration, such as the starting and stopping rules.

We defined the cluster score as the average item score within the cluster, such that the cluster score is on the same scale as the item scores and to allow for varying cluster sizes. Our results and procedures do not translate straightforwardly to other types of cluster scores, such as an “all items correct” or “at least one item correct” score. For such dichotomization of cluster scores, both the expected cluster score and the cluster response function are different, possibly leading to a different observed order of cluster scores.

In addition, we pursued a nonparametric IRT approach, such that no further restrictions were placed on the item or cluster response function besides the ordering structure, and the procedure made use of typical asymptotic tests for pairwise comparisons. Alternative procedures may be developed along the lines of, for instance, shift-scale parametric item response models (Haaf et al., 2020) or by incorporating resampling techniques such as permutation testing (e.g., Chung & Romano, 2013) for violations of pairwise manifest IIO or formulating Bayes factors for sets of order hypotheses (Van de Schoot et al., 2012).

The procedure for evaluating the ordering structure of a clustered item set may be used as part of a more elaborate scaling analysis, such as Mokken scale analysis (e.g., Mokken, 1971; Sijtsma & Molenaar, 2002; Sijtsma & Van der Ark, 2017). Other methods in this analysis provide insight in other test and item characteristics, such as the scalability of items, whether the item response functions are monotonically increasing, or whether there is local dependency between items. Furthermore, for a more systematic investigation of the construct validity, the proposed method may be complemented with the investigation of other item properties or attributes, such as by incorporating them into an explanatory (parametric) item response model as proposed by De Boeck and Wilson (2004, Section 1.4). In general, investigating the ordering structure of a test can provide useful information, but the decision to redesign or remove items or clusters should also be based on item content and theoretical considerations, to avoid losing important aspects of the theoretical construct in the measurement instrument.
